# 'MRI-negative PET-positive' temporal lobe epilepsy (TLE) and mesial TLE differ with quantitative MRI and PET: a case control study

**DOI:** 10.1186/1471-2377-7-16

**Published:** 2007-06-24

**Authors:** Ross P Carne, Terence J O'Brien, Christine J Kilpatrick, Lachlan R MacGregor, Lucas Litewka, Rodney J Hicks, Mark J Cook

**Affiliations:** 1 Victorian Epilepsy Centre, St Vincent's Hospital, Melbourne, Australia; 2 PET Centre, The Peter MacCallum Cancer Institute, Melbourne, Australia; 3 Department of Neurology, St. Vincent's Hospital, Melbourne, Australia; 4 Department of Neurology, The Royal Melbourne Hospital, Melbourne, Australia; 5 Department of Medicine, St. Vincent's Hospital, Melbourne, Australia; 6 Department of Medicine, The Royal Melbourne Hospital, Melbourne, Australia; 7 Department of Neuroscience, The Geelong Hospital, Barwon Health, Geelong, Australia; 8 Department of Clinical Epidemiology and Biostatistics, The Royal Melbourne Hospital, Melbourne, Australia; 9 School of Medicine, Deakin University, Geelong, Australia; 10 Faculty of Medicine, The University of Melbourne, Melbourne, Australia

## Abstract

**Background:**

'MRI negative PET positive temporal lobe epilepsy' represents a substantial minority of temporal lobe epilepsy (TLE). Clinicopathological and qualitative imaging differences from mesial temporal lobe epilepsy are reported. We aimed to compare TLE with hippocampal sclerosis (HS+ve) and non lesional TLE without HS (HS-ve) on MRI, with respect to quantitative FDG-PET and MRI measures.

**Methods:**

30 consecutive HS-ve patients with well-lateralised EEG were compared with 30 age- and sex-matched HS+ve patients with well-lateralised EEG. Cerebral, cortical lobar and hippocampal volumetric and co-registered FDG-PET metabolic analyses were performed.

**Results:**

There was no difference in whole brain, cerebral or cerebral cortical volumes. Both groups showed marginally smaller cerebral volumes ipsilateral to epileptogenic side (HS-ve 0.99, p = 0.02, HS+ve 0.98, p < 0.001). In HS+ve, the ratio of epileptogenic cerebrum to whole brain volume was less (p = 0.02); the ratio of epileptogenic cerebral cortex to whole brain in the HS+ve group approached significance (p = 0.06). Relative volume deficits were seen in HS+ve in insular and temporal lobes. Both groups showed marked ipsilateral hypometabolism (p < 0.001), most marked in temporal cortex. Mean hypointensity was more marked in epileptogenic-to-contralateral hippocampus in HS+ve (ratio: 0.86 vs 0.95, p < 0.001). The mean FDG-PET ratio of ipsilateral to contralateral cerebral cortex however was low in both groups (ratio: HS-ve 0.97, p < 0.0001; HS+ve 0.98, p = 0.003), and more marked in HS-ve across all lobes except insula.

**Conclusion:**

Overall, HS+ve patients showed more hippocampal, but also marginally more ipsilateral cerebral and cerebrocortical atrophy, greater ipsilateral hippocampal hypometabolism but similar ipsilateral cerebral cortical hypometabolism, confirming structural and functional differences between these groups.

## Background

Temporal lobe epilepsy is the commonest form of refractory focal epilepsy in adults. About 30% of temporal lobe epilepsy is due to foreign tissue lesions, whilst 60–70% is designated "non lesional" (NLTLE). The most common pathological substrate in this "non-lesional" group is hippocampal sclerosis (HS), present in a majority of medically refractory cases. Whilst it is difficult to give exact proportions, given that only patients with intractable seizures undergo full characterisation, around 70% of these patients with NLTLE are thought to have HS [1]. It is estimated that at least 30% of patients with NLTLE have neither a foreign tissue temporal lobe lesion nor MRI evidence of HS (HS-ve).

A previous study by our group demonstrated significant clinicopathological and qualitative structural and functional imaging differences between TLE associated with hippocampal sclerosis (HS+ve) and TLE with no evidence of hippocampal sclerosis on MRI (HS-ve) [2]. Apart from the lack of evidence of the defining hippocampal sclerosis (HS) on MRI, HS-ve patients in general showed lateralised but more widespread temporal hypometabolism on FDG-PET with blinded visual assessment. This group were therefore dubbed "MRI negative PET positive TLE". Other findings in the HS-ve group included a less frequent history of febrile convulsions; slower rhythms at ictal EEG onset; less frequent histopathological hippocampal sclerosis, and a similarly good post surgical outcome even in the subgroup of HS-ve patients who had undergone a hippocampal sparing procedure. Further study with Statistical Parametric Mapping (SPM) has suggested the main difference between the groups lies in greater hippocampal hypometabolism in the HS+ve group [3].

Brain volume deficits have been reported in epilepsies covering a range of syndromes and aetiologies, and to some extent can be used to differentiate these syndromes [4-6]. Temporal lobe epilepsy in particular has been associated with regional and more widespread areas of atrophic change, sometimes only evident with detailed volumetric study [7]. The most obvious structural difference between HS+ve and HS-ve temporal lobe epilepsy involves the presence or absence of hippocampal atrophy. However, an appreciation of more widespread differences in degree and distribution of volume deficits between these two groups, if present, may shed light on any possible pathophysiological differences.

The functional imaging characteristics of these groups are also of great interest. Many HS-ve patients, as with HS+ve patients, have prominent focal hypometabolism on FDG-PET scans [8], with rare false lateralisation. The underlying pathophysiological basis for the hypometabolism seen in patients with TLE is still unresolved. While FDG-PET has shown a high correlation with MRI-identified HS for the lateralisation of the epileptogenic zone, many studies have found that the magnitude of the hypometabolism correlates weakly or not at all with either direct [9] or indirect [10,11](MRI hippocampal volumetry) measures of hippocampal neuronal loss, even in patients with HS. In both HS-ve and HS+ve TLE, decreased metabolism may involve lateral as well as mesial temporal structures. Our previous study suggested more extensive hypometabolism in HS-ve patients compared to HS+ve on blinded visual assessment, extending to more commonly involve temporal structures beyond anterior or mesial regions [2]. However, the extent of differential involvement of temporal sub-regions and remote cortex on FDG-PET is difficult to quantitate visually.

Given the differences obvious on visual analysis, we were interested to investigate whether further differing patterns of structural or functional changes were present between these TLE subgroups using quantitative and semiquantitative methods. The primary hypothesis was that the epileptogenic focus in HS-ve patients involves primarily lateral rather than mesial temporal structures, and that the quantitative structural and functional changes would reflect this. The current study aimed to investigate this hypothesis by comparing volumetric MRI and coregistered FDG-PET metabolic measures on scans from HS+ve and HS-ve TLE patients.

## Methods

This study involved the same patient cohort as that from a previously reported case-control study [2], but on this occasion consisted of a detailed quantitative assessment of the patients' MRI and PET scans.

### Case Selection

Cases comprised 30 consecutive patients with clinically and video-EEG defined non-lesional temporal lobe epilepsy (NLTLE) and well-lateralised ictal EEG changes, but without evidence of HS on MRI, including MR volumetry (HS-ve). All patients had been admitted for a comprehensive inpatient assessment including video-EEG monitoring between 1996 and 2002 at one of three tertiary referral hospitals. Controls were 30 age- and sex-matched patients with well-lateralised unilateral ictal and/or interictal epileptiform EEG discharges and concordant unequivocal evidence of hippocampal sclerosis on MRI, confirmed by MR volumetry (HS+ve). The controls were selected by starting with the most recent HS+ve patient and moving consecutively retrospectively through the epilepsy monitoring database, matching as appropriate for age and sex. HS-ve patients represented close to 20% of medically refractory partial epilepsy patients assessed at these institutions. The study was approved by the institutional ethics committees of The Royal Melbourne Hospital, St. Vincent's Hospital and The Peter MacCallum Cancer Institute. All patients consented to MRI and PET scans as part of the study.

Historical features in these groups have been previously described [2]. Significantly there was no difference between the groups in duration of epilepsy, presence or type of auras, or pre-operative seizure frequency scores classified according to a 12-point seizure frequency score (SFS).

### MRI methods

All imaging was performed on a 1.5 Tesla GE Signa (GE Systems Milwaukee). In addition to standard axial and sagittal sequences, approximately 124 contiguous T1-weighted MPRAGE images were acquired, with FOV 24 cm, and a 256*256 matrix, providing in-plane resolution of 0.98 mm^2^. Resolution in the Z-plane was 1.5 mm. Images were obtained using a three-dimensional volume acquisition sequence in addition to standard sequences. Data was segmented, using a semi automated, three-dimensional morphometric protocol to define the structures of interest. The methods of MRI segmentation and quantitation have been previously described [12].

Cerebral cortex was extracted by an interactive thresholding method, performed by a single blinded observer, as described in a previous paper [12]. Hippocampi were measured according to a well defined protocol [13].

The groups were compared for MRI volumetric measures of: hippocampus; whole brain; total cerebral cortex; hemispheric volumes ipsilateral and contralateral to seizure onset; extracted hemispheric cortical volumes ipsi- and contralateral to seizure onset; and segmented lobar volumes of ipsi- and contralateral frontal, temporal, parietal, occipital and insular cortices.

### FDG-PET Methods

Patients were imaged in the interictal state on a PENN PET 300H Tomograph scanner with sodium iodide crystals, using a 25.6 cm diameter reconstruction field of view and 3D whole-head acquisition, according to a previously defined protocol [8]. For the 2 mm slice thickness used for whole-body imaging, the measured resolution was 4.2 mm at full width at half maximum transaxially and 5.4 mm at full width at half maximum out of plane. The dose of FDG administered was 37–111 MBq (1–3 mCi). One bed position was used. All scans were commenced 45 minutes after injection of FDG with the patient lying comfortably in a darkened room during the uptake period. The scan acquisition time was 30–40 minutes to achieve comparable statistical quality between scans. The duration of acquisition was based on count rates measured at the commencement of imaging, achieving total counts of >40 million. Measured, segmented attenuation correction was derived from a rotating 'single photon' Cs-137 source. The data were processed using a Wiener prefilter (scaling value = 0.5) and ordered-subsets expectation maximization (OSEM) iterative reconstruction performed with 4 iterations and 8 subsets. OSEM significantly reduces the degree of statistical noise in the reconstructed images compared to previous filtered back projection techniques on this system. The Wiener pre-filter improved resolution by enhancing the frequencies that define the resolving power of the system modulation transfer function (MTF). The images were reconstructed into a 256 × 250 mm cylindric volume with a 2 mm slice thickness. The reconstruction process created a standard series of contiguous images oriented in the transaxial, coronal, sagittal, and transtemporal planes.

[18F] FDG-PET scans were available for analysis in 30 HS-ve patients and 27 HS+ve patients. Three of the 27 FDG PET scans in the HS+ve group were technically suboptimal, and did not allow accurate coregistration to be performed. These three HS+ve FDG PET scans all showed lateralised ipsilateral temporal hypometabolism, and had been included in the previously published study reporting visual analyses (Carne et al. 2004). Coregistered FDG-PET analyses of these HS+ve controls and of the FDG PET scans of their matched MRI negative PET positive counterpart cases were not included in this analysis, allowing 27 HS-ve and 24 HS+ve patients to be analysed.

### Image Processing for MRI/FDG-PET coregistration

**Figure 1:**
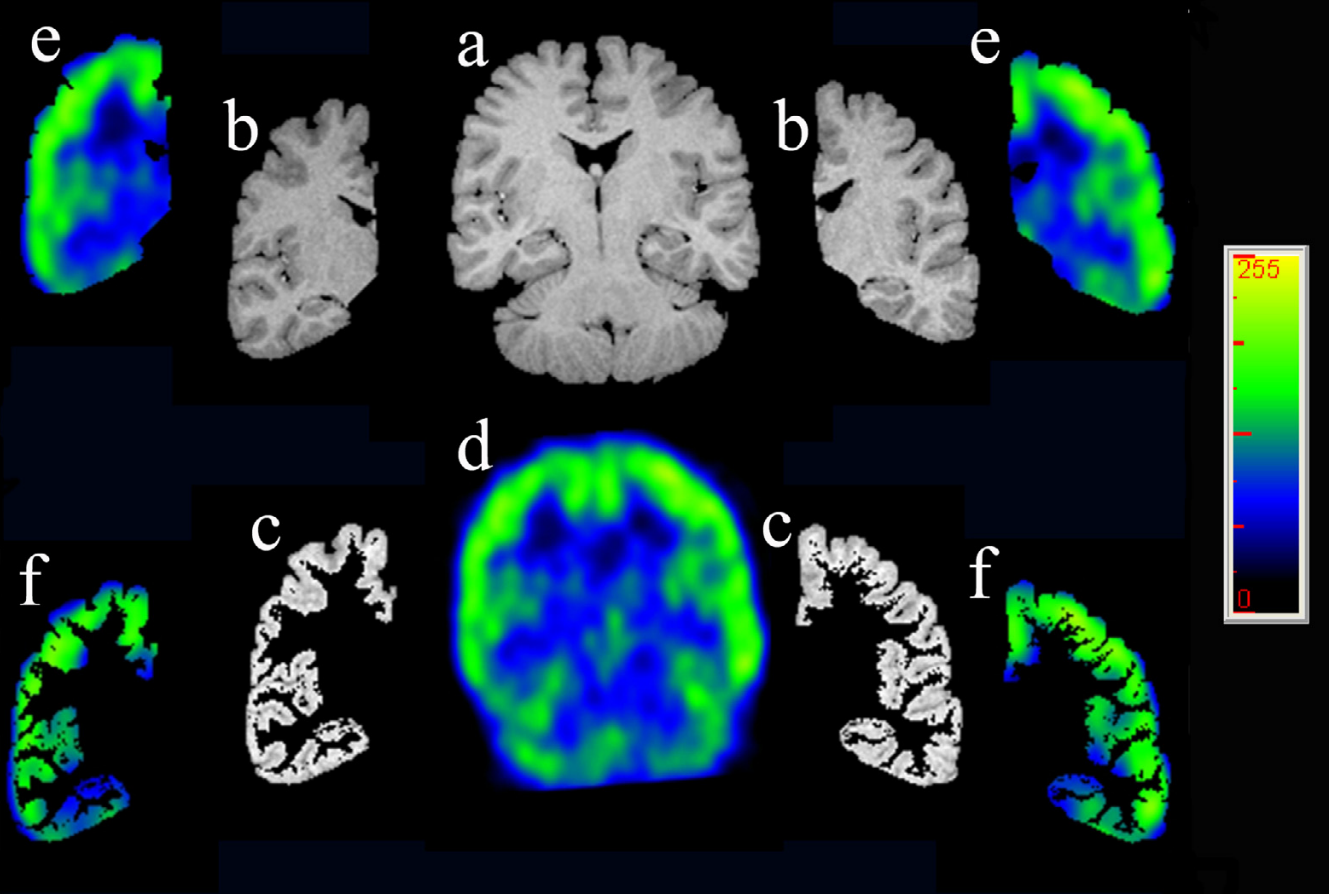
Volumetric MRI and coregistered FDG-PET: (a) MRI Extracted whole brain is segmented into (b) MRI right and left cerebral hemispheres, in turn thresholded into (c) MRI right and left cerebral cortices. Coregistration of the (d) FDG-PET whole brain allows accurate anatomical definition on the metabolic studies of, for example, (e) FDG-PET right and left cerebral hemispheres and (f) FDG-PET right and left cerebral cortices.

One of the difficulties with quantitation of regional changes on FDG-PET scans is the relatively lesser anatomical resolution obtained compared to MRI. To overcome this, the FDG-PET scan was coregistered with the patients volumetric MRI, and the detailed anatomical structures defined on the MRI used to accurately quantitate the associated metabolic change on the PET (Figure 1).

Coregistration was performed using a surface matching technique, involving conversion of MRI and FDG-PET to binary volumes and subsequent matching of surface points, using the AnalyzeAVW software package (Mayo Clinic). Binary images representing the cerebral surface for each scan were obtained by extracting the brain from extracerebral structures on MRI, as described previously [12], and thresholding out the extracerebral uptake on FDG-PET. Interior "holes" were then deleted, to give two binary single objects representing brain surface as measured using each modality.

**Figure 2:**
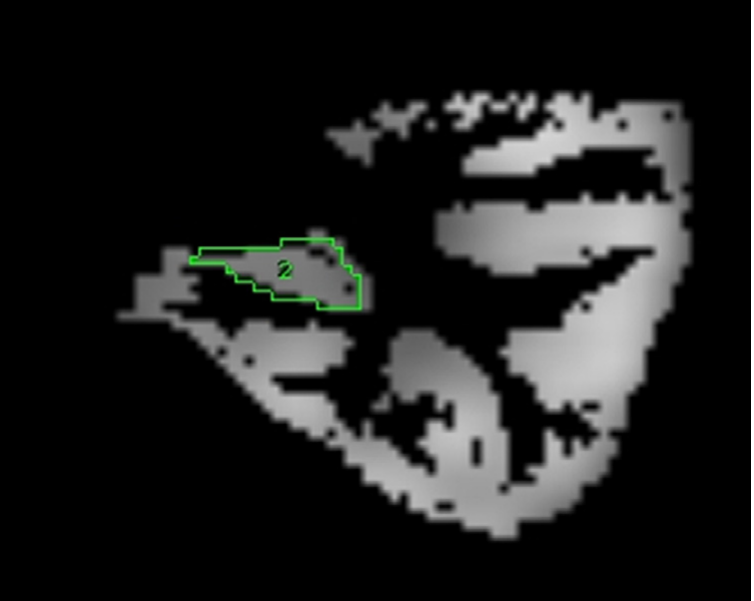
FDG-PET left temporal cortex with coregistered hippocampal tracing, coronal section.

One thousand points on the surface of the first cerebral binary image (PET) were sampled and the points then matched to the corresponding surface of the other cerebral binary image (MRI) resulting in a 4 × 4 matrix that best describes the three dimensional (3-D) transformation of the PET to the MRI image [Jiang H et al., 1992]. The calculated transformation matrix was then applied to the original interictal PET image, which resulted in an FDG-PET scan in the same 3-D space as the MRI. Structures or regions of interest then defined on MRI were then used to analyse the same areas on the associated PET, allowing a detailed structural-functional comparison to be made (Figure 1). The mean intensity on FDG-PET was then calculated in each of the areas defined at MRI segmentation: whole cerebrum, lateralised cerebral hemispheres, lateralised hemispheric cerebral cortex, hippocampus (Figure 2), and frontal, temporal, insular, parietal and occipital lobes (Figure 1). Further analysis was also performed of the temporal lobes with hippocampus, as defined by ROI on MRI, removed from the temporal cortex. The ratio of FDG uptake in ipsilateral (epileptogenic) to the homologous contralateral region was calculated, with a smaller ratio indicating a more marked degree of ipsilateral hypometabolism. Ratios of ipsi- and contralateral lobar to whole brain metabolism, and hippocampus to whole brain were performed to allow comparison of differential metabolic effects between lobes.

### Statistics

Analysis of ratios of ipsilateral epileptogenic to contralateral volumes was performed. The extent of volume asymmetry within each group was defined as the mean ipsilateral (epileptogenic)/contralateral side-to-side ratio. Brain volumes and PET intensity values were compared using either the paired t test, or Wilcoxon matched-pairs signed-ranks test where the distribution was clearly non-normal. Statistical analysis was performed using Stata Version 7.0 [14].

## Results

### MRI Brain Volume

Table 1 compares the results for the MRI derived volumes for the brain regions between the two groups. There were no significant differences in mean whole brain volume between groups (p = 0.56).

**Table 1:** Hippocampal, cerebral hemispheric and cerebrocortical hemispheric volumes: epileptogenic hemisphere "ipsilateral" and hemisphere "contralateral" to seizure onset.

**MRI volumetry (cm3)**	**HS+ve n = 30**		**HS-ve n = 30**	
	**Mean**	**Standard deviation**	**Mean**	**Standard Deviation**
**Total Cerebral**	1,230	124.3	1,256	267.2
**Ipsilateral Cerebral**	527.8	55.63	543.9	117.9
**Contralateral Cerebral**	536.5	53.52	547.4	118.1
**Total Cerebral Cortex**	628.3	73.62	613.2	138.2
**Ipsilateral Cerebral Cortex**	312.3	38.31	307.5	69.80
**Contralateral Cerebral Cortex**	315.7	35.88	305.4	68.80
**Ipsilateral hippocampus**	2.845	0.641	4.087	0.889
**Contralateral Hippocampus**	4.091	0.576	4.044	0.881

**Figure 3:**
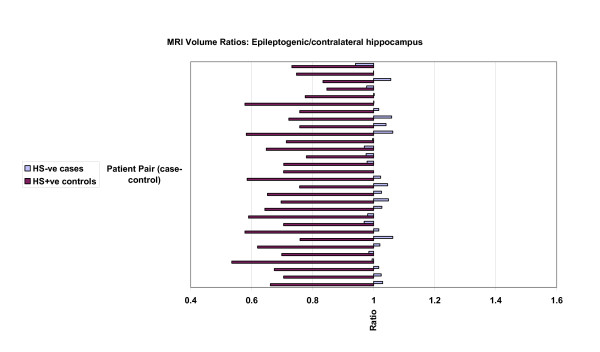
MRI volumetric comparisons: HS-ve versus HS+ve. Case-control pairs are presented in order of ascending age. Ratios presented relate to epileptogenic/contralateral MRI volumes: hippocampus. Mean and standard deviation figures are given in Table 2.

**Figure 4:**
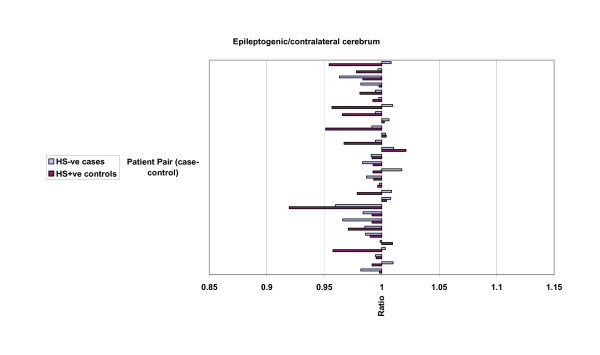
MRI volumetric comparisons: HS-ve versus HS+ve. Case-control pairs are presented in order of ascending age. Ratios presented relate to epileptogenic/contralateral MRI volumes: cerebrum. Mean and standard deviation figures are given in Table 2.

**Figure 5:**
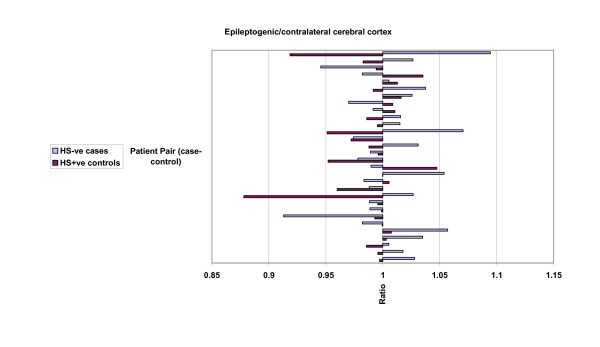
MRI volumetric comparisons: HS-ve versus HS+ve. Case-control pairs are presented in order of ascending age. Ratios presented relate to epileptogenic/contralateral MRI volumes: cerebral cortex. Mean and standard deviation figures are given in Table 2.

The results of analysis of mean hippocampal, cerebral hemispheric and cerebrocortical hemispheric ratios of ipsilateral (epileptogenic) side to contralateral volume ratios are presented in table 2, and graphically in figures 3, 4 and 5. Lower ratios indicate greater ipsilateral atrophy. The HS+ve group had marked hippocampal atrophy (ratio 0.69, p < 0.001), but also were found to have a small but significant relative ipsilateral cerebral hemispheric atrophy (0.98, p < 0.001). Hippocampal volumes in the HS-ve group, by definition, were relatively symmetrical. Cerebral cortical volumes in the HS-ve group were also relatively symmetrical, although there was a detectable minor deficit in ipsilateral cerebral hemispheric volume (0.99, p = 0.018). When both populations were compared on a case-control basis, there was not only greater hippocampal atrophy (p < 0.001) but also a marginally greater degree of ipsilateral cerebral (p = 0.022) and possibly cerebrocortical (p = 0.06) atrophy in the HS+ve group.

**Table 2:** Ratios of ipsilateral (epileptogenic) to contralateral volumes.

	HS+ve mean (SD)	p	HS-ve mean (SD)	**p**	**Between Groups:**
**Hippocampi.**	0.69 (0.08)	<0.001	1.01 (0.18)	0.08	p < 0.001
**Cerebrum.**	0.98 (0.02)	<0.001	0.99 (0.18)	0.018	p = 0.022
**Cerebral Cortex.**	0.99 (0.03)	0.08	1.01 (0.19)	0.37	p = 0.06

Key Table 2. The p-value quoted relates to side-to-side comparisons within individuals across the group. The between group comparison relates to comparison of the case to the age and sex matched control, performed across the group, rather than being a population comparison of ipsilateral to contralateral mean volumes. These data are presented graphically in Figure 3.

Specific measures of hippocampal volume ratios between the groups were of interest. While the mean volume of the smaller hippocampus in the HS+ve group (2845 mm3) differed from the smaller hippocampus in the HS-ve group (4013 mm3, p < 0.001), there was no detectable difference in the size of the larger hippocampus between groups (HS+ve 4091 mm3; HS-ve 4121 mm3, p = 0.83). Similarly, there was no detectable difference in the mean volume of the hippocampus contralateral to seizure onset between groups (HS+ve 4091 mm3; HS-ve 4044 mm3; p = 0.73).

The absolute cortical lobar volumes and ipsilateral/contralateral ratios are shown in Tables 3 and 4. HS+ve showed smaller ipsilateral volumes across all lobes except frontal, however only insular and temporal comparisons approached statistical significance. HS-ve ipsilateral volumes were in contrast relatively similar to or larger than contralateral volumes. (Table 4).

**Table 3:** Cortical lobar volumes ipsilateral and contralateral to seizure onset.

**MRI Volumetry (cm3)**	**HS+ve**				**HS-ve**			
	**Ipsilat**		**Contralat**		**Ipsilat**		**Contralat**	
	**Mean**	**SD**	**Mean**	**SD**	**Mean**	**SD**	**Mean**	**SD**
**Frontal Cortex**	114.7	15.91	113.6	15.77	110.5	25.07	110.7	25.30
**Temporal Cortex**	79.28	11.70	81.441	10.64	79.11	20.28	78.49	19.78
**Parietal Cortex**	85.78	13.99	86.44	12.76	85.83	22.09	82.34	20.34
**Occipital Cortex**	26.34	6.738	25.77	6.386	25.48	7.290	25.72	6.690
**Insular Cortex**	7.160	1.242	7.500	1.029	7.168	1.670	7.080	1.794

**Table 4:** Ratios of ipsilateral (epileptogenic) to contralateral volumes.

	**HS+ve **mean (SD)	**p**	**HS-ve **mean (SD)	**p**	**Between groups**
**Frontal**	1.01 (0.26)	0.19	1.00 (0.25)	0.82	p = 0.24
**Temporal**	0.98 (0.26)	0.09	1.01 (0.20)	0.60	p = 0.18
**Parietal**	0.99 (0.26)	0.63	1.04 (0.21)	0.016	p = 0.08
**Occipital**	0.97 (0.27)	0.32	0.99 (0.27)	0.67	p = 0.84
**Insular**	0.95 (0.11)	0.035	1.03 (0.22)	0.60	p = 0.039

Key Tables 4-7. The p-value quoted relates to side-to-side comparisons within individuals across the group. The between group comparison relates to comparison of the case to the age and sex matched control, performed across the group, rather than being a population comparison of ipsilateral to contralateral mean volumes.

### Quantitative FDG-PET Analysis

The results of blinded visual analysis in these two groups of patients were previously described, with clearly lateralised hypometabolism in the majority of both groups: 27/30 HS-ve patients and 100% of HS+ve patients in whom PET was performed (27/27) [2]. Where lateralised, the side of the PET hypometabolism was concordant with that of the ictal EEG in 26/27 HS-ve and with ictal EEG and MRI in all HS+ve patients.

Semi-quantitative analysis of hypometabolism using coregistration confirmed that mesial (hippocampal) ipsilateral hypometabolism, although present in both HS+ve (ipsi/contralateral ratio 0.86) and HS-ve (ipsi/contralateral ratio 0.95), was more marked in the HS+ve group (p < 0.001). There was no significant correlation between the degree of MRI determined volume loss and FDG-PET determined metabolic asymmetry in either group (correlation coefficients: HS+ve R = 0.26, p > 0.05; HS-ve R = 0.23, p > 0.05): this is particularly obvious in the HS-ve group where greater ipsilateral/contralateral cortical (1.01) and hippocampal (1.01) volume ratios contrasted with markedly lesser ipsilateral/contralateral metabolic ratios (0.97 and 0.95 respectively). (Table 5).

**Table 5:** FDG-PET mean intensity: Cerebrum, Cerebral cortex and Hippocampi. Ipsilateral/Contralateral Ratios: lower ratios indicate greater ipsilateral hypometabolism.

	**HS+ve **mean (SD) **n = 24**	**p**	**HS-ve **mean (SD) **n = 27**	**p**	**Between Groups:**
**Hippocampi**	0.86 (0.09)	<0.001	0.95 (0.06)	<0.001	p < 0.001
**Cerebrum.**	0.98 (0.02)	<0.001	0.97 (0.18)	<0.001	p = 0.107
**Cerebral Cortex.**	0.98 (0.03)	0.002	0.97 (0.18)	<0.001	p = 0.363

In contrast to the more marked mesial hypometabolism in the HS+ve group, lateralised cerebral and cerebrocortical hypometabolism, although again present in both HS+ve (ipsi/contralateral ratio 0.98) and HS-ve (ipsi/contralateral ratio 0.97) groups, were similar in the two groups; if anything, less in the HS-ve group although the difference was not statistically significant: (cerebral p = 0.107; cerebrocortical p = 0.363).

Comparing these results to the MRI volumetric results yielded interesting data. The magnitudes of the volume and metabolic asymmetries showed no significant correlation (R = 0.25, p > 0.05 in both groups). The magnitude of the side-to-side hippocampal FDG-PET ratio was significantly less in the HS+ve group (HS+ve:0.86+/-0.08, HS-ve:0.95+/-0.18, p < 0.001). On blinded visual analysis the side of the FDG-PET hypometabolism concurred with EEG side in all HS+ve and 25/27 HS-ve patients [2]. The side of hippocampal hypometabolism by ROI analysis concurred with EEG side in all HS+ve but only 22/27 HS-ve patients, suggesting that in some HS-ve cases it is the changes beyond the hippocampus that allow lateralisation of the epileptogenic temporal lobe, rather than ipsilateral hippocampal hypometabolism.

Results of cortical ipsilateral/contralateral lobar metabolic ratios are presented in Tables 6 and 7: lower values again indicate greater ipsilateral hypometabolism. The striking finding is that overall in both groups all ipsilateral lobes were comparatively hypometabolic, except for occipital lobe bilaterally where the difference was not significant (HS+ve, p = 0.84; HS-ve p = 0.069), and for HS+ve parietal lobe (0.98, p = 0.095). The only area where HS+ve showed greater ipsilateral compared to contralateral hypometabolism than HS-ve was in hippocampi (0.86 HS+ve vs 0.95 HS-ve, p < 0.001) In all other lobar regions, HS-ve patients showed similar or greater ipsilateral hypometabolism, confirming the visual impression of more widespread and more marked hypometabolism in the HS-ve group. Interestingly, there was also a suggestion of more overall temporal cortical hypometabolism ipsilaterally in HS-ve, in spite of the more significant mesial temporal hypometabolism in the HS+ve group (temporal cortex 0.94 HS+ve vs 0.92 HS-ve, p = 0.111).

**Table 6:** FDG-PET mean intensity: Cerebral cortical lobes. Ipsilateral/Contralateral Ratios: lower ratios indicate greater ipsilateral hypometabolism.

	**HS+ve **mean (SD)	**p**	**HS-ve **mean (SD)	**p**	**Between Groups: p**
**Frontal Cortex**	0.98 (0.35)	0.032	0.98 (0.25)	0.009	0.818
**Temporal Cortex**	0.94 (0.31)	<0.001	0.92 (0.24)	<0.001	0.111
**Parietal Cortex**	0.98 (0.35)	0.095	0.97 (0.25)	<0.001	0.113
**Occipital Cortex**	1.00 (0.36)	0.844	0.98 (0.25)	0.069	0.202
**Insular Cortex**	0.94 (0.06)	<0.001	0.95 (0.19)	0.001	0.456
**Hippocampi**	0.86 (0.09)	<0.001	0.95 (0.06)	<0.001	<0.001
**Temporal Cortex, hippocampi excluded.**	0.94 (0.30)	<0.001	0.92 (0.18)	0.196	0.135

**Table 7:** FDG-PET mean intensity: Cerebral cortical lobar ratios to whole brain mean: lower ratios indicate greater hypometabolism. WBM: whole brain mean; ipsi = ipsilateral; contra = contralateral.

**Ratios to Whole Brain Mean FDG-PET intensity.**	**HS+ve**		**HS-ve**	
	**Ipsi/WBM **mean (SD)	**Contra/WBM **mean (SD)	**Ipsi/WBM **mean (SD)	**Contra/WBM **mean (SD)
**Frontal Cortex**	1.123 (0.40)	1.145 (0.41)	1.11 (0.28)	1.13 (0.29)
**Temporal Cortex**	0.934 (0.30)	0.987 (0.32)	0.91 (0.24)	0.99 (0.25)
**Parietal Cortex**	1.135 (0.35)	1.150 (0.40)	1.11 (0.28)	1.14 (0.29)
**Occipital Cortex**	1.136 (0.41)	1.133 (0.41)	1.14 (0.29)	1.16 (0.30)
**Insular Cortex**	1.044 (0.07)	1.099 (0.07)	1.03 (0.20)	1.09 (0.21)
**Temporal Cortex, hippocampi excluded**	0.941 (0.39)	0.995 (0.41)	0.97 (0.25)	0.95 (0.19)
**Hippocampus**	0.692 (0.07)	0.791 (0.07)	0.760 (0.15)	0.801 (0.17)

## Discussion

### MRI Volumetric studies

Brain volume abnormalities have been reported in epilepsies ranging from primary generalised epilepsies through to lesional epilepsies [4-6]. There are also reports of progressive hippocampal volume deficits in patients with hippocampal sclerosis [15-17], of progressive hippocampal atrophy following status epilepticus [18], of progressive neocortical damage in chronic partial epilepsies [19], and of progressive temporal lobe atrophy following temporal lobectomy for intractable seizures [20]. Although these reports associate the atrophic process with epilepsy pathogenesis, it remains unclear whether these changes are primary or secondary.

The distribution of atrophy seems relatively specific for particular epilepsy syndromes [21,22]. In a study of cortical volumes in primary generalised epilepsy patients, patients had significantly larger cortical grey matter volumes than control subjects [5], most marked in patients with juvenile myoclonic epilepsy in mesial frontal lobes [23]. This is in contrast to the atrophy usually detected in partial epilepsy studies. Hippocampal atrophy is the most obvious feature on structural MRI in patients with hippocampal sclerosis [13], however other features of regional atrophy seem specific to temporal lobe epilepsy associated with HS. Atrophy of the entorhinal and perirhinal cortex have been shown to be present in MTLE and not in other conditions such as extratemporal or primary generalised epilepsies [24]. Even within the atrophic hippocampus in MTLE there is a predilection for some regions over others: the hippocampal head is more atrophic than the hippocampal body and hippocampal tail [25], while within the parahippocampal region, the entorhinal cortex is more severely affected than the perirhinal cortex [26-28]. Sophisticated hippocampal mapping techniques also show differences between the ipsi- and contralateral hippocampi in the regions most markedly affected, with marked inward deviation in the Sommer sector of the MTS hippocampi, and subicular but minimal Sommer sector involvement in the contralateral hippocampus [29].

Past studies of MRI negative TLE have not shown the consistent widespread volume deficits associated with hippocampal atrophy [30]. Extratemporal focal epilepsies more often have been shown to have demonstrable volume loss involving the predominant region or lobe affected [31,32]. In the study by Briellmann et al, hemicranial volume ipsilateral to the epileptogenic side was smaller in temporal lobe epilepsy patients, both with and without histopathologically proven HS, however ipsilateral atrophy was more marked in the patients with HS, a finding which our study has confirmed [32].

This study is the first to our knowledge examining volume deficits across segmented cortical lobes in a large group of TLE patients with MRI normal to visual inspection. Questions have remained as to whether this group has histopathological HS without detectable hippocampal atrophy or T2 signal changes on MRI, or may have bilateral HS making detection difficult on volumetric imaging. It can be assumed from past studies that there are varyingly subtle regional volume deficits in the HS+ve group, ranging from obvious hippocampal deficits through to subtle hemicerebral or whole brain abnormalities. The finding of small but significant differences in ipsilateral cerebral and cerebrocortical hemispheric volumes between the HS+ve and HS-ve groups is further evidence of a difference between these two groups, with a locoregional difference in emphasis in structures most involved.

Analysis by lobar subdivisions underlined these regional differences. Comparison of ipsilateral to contralateral lobar cerebral cortical volumes suggested that all lobes in HS+ve patients were smaller ipsilaterally except for frontal cortex. Atrophy of whole cerebral cortex on the ipsilateral side was small overall (at 0.99 ipsilateral/contralateral ratio), and this difference was accounted for by differences across most lobes. Greatest differences were seen in insular (0.95) and occipital (0.97) lobes, followed by temporal (0.98) and parietal lobes (0.99). The reported ratio represents the mean ratio across the group; the p value relates to a paired samples comparison of ipsilateral versus contralateral volume differences within the same patient across the group, hence the most significant differences do not necessarily correspond with the lowest mean ratios: insular (p = 0.035) cortical atrophy was most marked here, with temporal cortical atrophy (p = 0.09) approaching significance.

HS-ve patients interestingly and contrastingly showed larger ipsilateral/contralateral ratios in all lobes except occipital (0.99, p = 0.67), with parietal cortex most markedly larger ipsilaterally (1.04, p = 0.016). This contrasts also with the finding of whole ipsilateral cerebral hemispheric atrophy (0.99), suggesting that the mild ipsilateral cerebral volume loss in the HS-ve group relates to white matter rather than cortical volume deficit.

The unexpected finding of larger ipsilateral parietal cortex in the HS-ve group is perhaps a chance finding relating to use of multiple comparisons, although the finding of larger cortical volumes in patients with epilepsy does have precedent in the primary generalized epilepsy literature [23]. Next most marked in the between group differences was the insular comparison, with the ipsilateral atrophy in the HS+ve group (0.95) markedly greater than in the HS-ve group (1.03) (p = 0.039): this is supported by the known involvement of insular cortex in mesial temporal lobe epilepsy [33]. Temporal cortical lobar atrophy was also possibly more marked in the HS+ve group (0.98 vs 1.01 HS-ve, p = 0.11). These findings support the contention that HS+ve extends to involve structures distant from the mesial temporal lobe, with more widespread evidence of cortical atrophy, and also that HS+ve differs from HS-ve in the site of predominant involvement, with relatively preserved cortical volumes but hemicranial volume deficits in the HS-ve group.

### Coregistration FDG-PET Regional Quantitations

The striking finding of ipsilateral hypometabolism in both groups in virtually all brain regions confirms the impression of a widespread functional deficit in patients in both groups of non lesional TLE. Although differences are present, the similarities between these groups are also interesting. Both groups have most marked hypometabolism in the temporal lobes, maximal mesially in the HS+ve group. Beyond the temporal lobes, hypometabolism was similar, although in the HS-ve group this trended towards being more marked (e.g. parietal 0.97 HS-ve vs 0.98 HS+ve, p = 0.084): a small difference if present, but a marked contrast to the mesially focussed hypometabolism in the HS+ve group (0.95 HS-ve vs 0.86 HS+ve, p < 0.001). Exclusion of hippocampi confirmed the impression that hippocampi bear the brunt of the functional deficit in HS+ve [3], but are also involved in HS-ve.

A potential limitation of this study involves the use of multiple comparisons, and it is possible that with multiple statistical tests that some of the results will arise by chance. In this situation it is appropriate, however, to interpret data based on the primary hypothesis, in this case that the epileptogenic focus in HS-ve patients involves primarily lateral rather than mesial temporal structures, and that the quantitative structural and functional changes would reflect this: that ipsilateral atrophy and hypometabolism would be present, and that the extent of hypometabolism would be greater in HS-ve, where the focus was posited to be lateral rather than mesial. The data presented relate either to structural or metabolic asymmetry and deal directly with this primary hypothesis. The reporting of supportive results in combination with a constellation of low or trending-low p values in support of this theory is compelling.

In both HS-ve and HS+ve groups the percentage of patients with lateralised concordant hypometabolism on FDG PET scanning was high, both by visual and semi quantitative analyses. In a previous study, the extent of hypometabolism on PET was found to extend more frequently beyond anterior and or mesial temporal regions alone to involve other temporal or extratemporal regions in the HS-ve group [2]. Other features of the HS-ve group suggested that the focus in the group of patients does not lie mesially, but is more likely to involve the lateral temporal neocortex.

Other groups have noted the difference in the FDG PET findings between temporal lobe epilepsy patients with primarily mesial and lateral foci. One study [34] found significant differences between mesial and lateral TLE in similar pattern: that study included 35 patients with lateral TLE, only 11 of whom were non lesional, the remainder of whom had CD, vascular malformations or tumours. They found that hypometabolism was more prominent in the lateral structures than mesial in both groups, which differs from our finding in the mesial group with proven hippocampal sclerosis who had more marked mesial hypometabolism, a finding also reported by other groups [35]. It is interesting though to compare the findings in the lateral group in that study, where the majority had observed structural lesions – the best 'gold standard' for seizure onset localization – with our study where structural lesions were not evident. This similar pattern of hypometabolic involvement in our non lesional group suggests a lateral temporal onset in the HS-ve patients.

This striking mesial/hippocampal emphasis in the HS+ve group suggests that this group may be relatively homogenous in its involvement of mesial structures/hippocampus, whereas there is no corresponding single structure or area that is consistently involved in the HS-ve group. This raises the question as to whether the non lesional group is a disease, a syndrome, or a collection of unrelated entities that have in common no clear epileptogenic lesion on the MRI. The temptation to define this group as temporal lobe epilepsy that is not "HS" can to some extent be countered by the findings on FDG-PET, a positive feature for definition that clearly differentiates the group from extratemporal epilepsies, and the MRI, which differentiates the group from MTLE. Beyond this it is possible that HS-ve temporal lobe epilepsy represents a group with diverse aetiologies, but on balance highly unlikely given the overall similarities otherwise on structural, functional and histopathological comparisons.

The pathophysiology underlying the hypometabolism seen on FDG PET in temporal lobe epilepsy remains uncertain, and although predictably observed and well described is not well explained by observed macro or microstructural alterations. The area of hypometabolism on FDG PET is generally thought much larger than the area of presumed pathological involvement [36], though the widespread extent of both hypometabolism and cortical atrophy in both groups is confirmed by our study. Electroclinical studies suggest that the pattern of hypometabolism may relate to both the onset of the ictal discharge and to the patterns of preferential spread [37]. A relationship between interictal slow wave activity and FDG PET hypometabolism may indicate that the hypometabolic area represents a region of increased neuronal inhibition which can receive interictal and ictal propagation [38]. Quantitative FDG-PET uptake studies have shown the most marked deficits when comparing HS+ve TLE patients to controls bilaterally in frontal and parietal lobes, in comparison to the more marked abnormalities detected in temporal lobes with asymmetry indices [39]. There is conflicting evidence as to whether the extent or severity of hypometabolism relates to neuronal loss [40,9,11], duration of prior habitual seizures [41,42] or severity of seizures [43]. In our study, the finding of significant mesial hypometabolism in the MRI negative (HS-ve) group, despite volumetrically normal hippocampi, is evidence against the contention that volume loss and hypometabolism are significantly correlated. This also argues against partial volume effect being a major contribution to the results at the hippocampal level. Nevertheless, this contribution cannot be excluded, and is a potential limitation of the study. Further analysis with partial volume correction, or alternatively with voxel based morphometric methods to more closely delineate sites of structural or metabolic heterogeneity may help to clarify this further. Even in our HS+ve group, there was not a significant correlation between degrees of MRI volumetric and FDG-PET metabolic asymmetry (correlation coefficient 0.26). The fact that hypometabolism is independent of neuronal loss perhaps accounts for it being an independent predictor of surgical outcome [44-46].

Another potential limitation of the study is that we did not assess for changes in smaller subregions of the brain. The large structures assessed (i.e. hemispheres) are heterogeneous and therefore focal changes at lobar and sublobar levels could easily have been missed. However, the limited number of subjects that were available for inclusion in this study did not allow the statistical power to assess multiple regions of interest. Future studies, involving larger numbers of patients, should extend from our results by investigating for regional changes particularly in the cortex.

The smaller hemicranial volumes and varying but almost ubiquitous hypometabolism on the side of the epileptogenic focus occurring irrespective of the presence or absence of HS are of great interest. Does this represent a differing pathological process in the hemisphere to that in the hippocampus, or is this the same process with a difference in regional emphasis? There is little or no structural histopathological evidence to suggest that the associated findings in the lateral neocortex and hemispheric structures differ significantly between the groups; given this, perhaps the latter hypothesis seems the most valid. Such differences in location of primary involvement may derive from differential timing of an initial insult: for instance given differential embryological timing of hippocampal and adjacent neocortical development, it is conceivable that a precisely timed embryological insult could affect one region with relative sparing of the other; with the subsequent response of surviving cortex determining the degree of the eventual predilection towards epileptogenesis, an interesting hypothesis that will require further study.

## Conclusion

Overall, HS+ve patients showed more hippocampal, but also marginally more ipsilateral cerebral and cerebrocortical atrophy, greater ipsilateral hippocampal hypometabolism but similar ipsilateral cerebral cortical hypometabolism, confirming structural and functional differences between these groups, in support of the clinical findings which suggest that these two groups represent differing syndromes.

## Competing interests

The author(s) declare that they have no competing interests.

## Authors' contributions

All authors were involved in manuscript revision. All authors read and approved the final manuscript.

RC participated in study design, coordinated the study, collated the data, participated in image manipulation and analysis and drafted the manuscript.

TOB participated in study conception, coordination and editing of the manuscript.

CK participated in study conception, coordination and editing of the manuscript

LM participated in study design and performed the statistical analysis.

LL helped substantially with the image manipulation, segmentation and analysis.

RH helped with PET data acquisition and data analysis.

MC participated in study conception, coordination and editing of the manuscript.

## Pre-publication history

The pre-publication history for this paper can be accessed here:



## References

[B1] Cascino GD, Jack CR, Parisi JE, Sharbrough FW, Hirschorn KA, Meyer FB, Marsh WR, O'Brien PC (1991). Magnetic resonance imaging-based volume studies in temporal lobe epilepsy: pathological correlations. Ann Neurol.

[B2] Carne RP, O'Brien TJ, Kilpatrick CJ, MacGregor LR, Hicks RJ, Murphy MA, Bowden SC, Kaye AH, Cook MJ (2004). MRI-negative PET-positive temporal lobe epilepsy: a distinct surgically remediable syndrome. Brain.

[B3] Carne RP, Cook MJ, MacGregor LR, Kilpatrick CJ, Hicks RJ, O'Brien TJ (2006). 'MRI negative PET positive' Temporal Lobe Epilepsy (TLE): FDG-PET pattern differs from mesial TLE. Mol Imaging and Biol.

[B4] Sisodiya SM, Free SL, Stevens JM, Fish DR, Shorvon SD (1997). Widespread cerebral structural changes in two patients with gelastic seizures and hypothalamic hamartomata. Epilepsia.

[B5] Woermann FG, Sisodiya SM, Free SL, Duncan JS (1998). Quantitative MRI in patients with idiopathic generalized epilepsy. Evidence of widespread cerebral structural changes. Brain.

[B6] Marsh L, Morrell MJ, Shear PK, Sullivan EV, Freeman H, Marie A, Lim KO, Pfefferbaum A (1997). Cortical and hippocampal volume deficits in temporal lobe epilepsy. Epilepsia.

[B7] Bonilha L, Rorden C, Halford JJ, Eckert M, Appenzeller S, Cendes F, Li LM (2006). Asymmetrical extra-hippocampal gray matter loss related to hippocampal atrophy in patients with medial temporal lobe epilepsy. J Neurol Neurosurg Psychiatry.

[B8] O'Brien TJ, Hicks RJ, Ware R, Binns DS, Murphy M, Cook MJ (2001). The utility of a 3-dimensional, large-field-of-view, sodium iodide crystal – based PET scanner in the presurgical evaluation of partial epilepsy. J Nucl Med.

[B9] Henry TR, Babb TL, Engel J, Mazziotta JC, Phelps ME, Crandall PH (1994). Hippocampal neuronal loss and regional hypometabolism in temporal lobe epilepsy. Ann Neurol.

[B10] Semah F, Baulac M, Hasboun D, Frouin V, Mangin JF, Papageorgiou S, Leroy-Willig A, Philippon J, Laplane D, Samson Y (1995). Is interictal temporal hypometabolism related to mesial temporal sclerosis? A positron emission tomography/magnetic resonance imaging confrontation. Epilepsia.

[B11] O'Brien TJ, Newton MR, Cook MJ, Berlangieri SU, Kilpatrick C, Morris K, Berkovic SF (1997). Hippocampal atrophy is not a major determinant of regional hypometabolism in temporal lobe epilepsy. Epilepsia.

[B12] Carne RP, Vogrin S, Litewka L, Cook MJ (2006). Cerebral cortex: an MRI based study of volume and variance with age and sex. J Clin Neurosci.

[B13] Cook MJ, Fish DR, Shorvon SD, Straughan K, Stevens JM (1992). Hippocampal volumetric and morphometric studies in frontal and temporal lobe epilepsy. Brain.

[B14] College Station TSC (2001). Stata Statistical Software.

[B15] Briellmann RS, Berkovic SF, Syngeniotis A, King MA, Jackson GD (2002). Seizure-associated hippocampal volume loss: a longitudinal magnetic resonance study of temporal lobe epilepsy. Ann Neurol.

[B16] Liu RS, Lemieux L, Bell GS, Bartlett PA, Sander JW, Sisodiya SM, Shorvon SD, Duncan JS (2001). A longitudinal quantitative MRI study of community-based patients with chronic epilepsy and newly diagnosed seizures: methodology and preliminary findings. Neuroimage.

[B17] O'Brien TJ, So EL, Meyer FB, Parisi JE, Jack CR (1999). Progressive hippocampal atrophy in chronic intractable temporal lobe epilepsy. Ann Neurol.

[B18] Wieshmann UC, Woermann FG, Lemieux L, Free SL, Bartlett PA, Smith SJ, Duncan JS, Stevens JM, Shorvon SD (1997). Development of hippocampal atrophy: a serial magnetic resonance imaging study in a patient who developed epilepsy after generalized status epilepticus. Epilepsia.

[B19] Liu RS, Lemieux L, Bell GS, Hammers A, Sisodiya SM, Bartlett PA, Shorvon SD, Sander JW, Duncan JS (2003). Progressive neocortical damage in epilepsy. Ann Neurol.

[B20] Ellamushi H, Moran NF, Kitchen ND, Stevens JM, Kendall BE, Lemieux L (2000). Generalised cerebral atrophy following temporal lobectomy for intractable epilepsy associated with mesial temporal sclerosis. Magn Reson Imaging.

[B21] Gartner B, Seeck M, Michel CM, Delavelle J, Lazeyras F (2004). Patients with extratemporal lobe epilepsy do not differ from healthy subjects with respect to subcortical volumes. J Neurol Neurosurg Psychiatry.

[B22] Natsume J, Bernasconi N, Andermann F, Bernasconi A (2003). MRI volumetry of the thalamus in temporal, extratemporal, and idiopathic generalized epilepsy. Neurology.

[B23] Woermann FG, Free SL, Koepp MJ, Sisodiya SM, Duncan JS (1999). Abnormal cerebral structure in juvenile myoclonic epilepsy demonstrated with voxel-based analysis of MRI. Brain.

[B24] Bernasconi N, Andermann F, Arnold DL, Bernasconi A (2003). Entorhinal cortex MRI assessment in temporal, extratemporal, and idiopathic generalized epilepsy. Epilepsia.

[B25] Hogan RE, Bucholz RD, Choudhuri I, Mark KE, Butler CS, Joshi S (2000). Shape analysis of hippocampal surface structure in patients with unilateral mesial temporal sclerosis. J Digit Imaging.

[B26] Bernasconi N, Bernasconi A, Caramanos Z, Antel SB, Andermann F, Arnold DL (2003). Mesial temporal damage in temporal lobe epilepsy: a volumetric MRI study of the hippocampus, amygdala and parahippocampal region. Brain.

[B27] Bonilha L, Kobayashi E, Rorden C, Cendes F, Li LM (2003). Medial temporal lobe atrophy in patients with refractory temporal lobe epilepsy. J Neurol Neurosurg Psychiatry.

[B28] Jutila L, Ylinen A, Partanen K, Alafuzoff I, Mervaala E, Partanen J, Vapalahti M, Vainio P, Pitkanen A (2001). MR volumetry of the entorhinal, perirhinal, and temporopolar cortices in drug-refractory temporal lobe epilepsy. AJNR Am J Neuroradiol.

[B29] Hogan RE, Wang L, Bertrand ME, Willmore LJ, Bucholz RD, Nassif AS, Csernansky JG (2004). MRI-based high-dimensional hippocampal mapping in mesial temporal lobe epilepsy. Brain.

[B30] Woermann FG, Free SL, Koepp MJ, Ashburner J, Duncan JS (1999). Voxel-by-voxel comparison of automatically segmented cerebral gray matter – A rater-independent comparison of structural MRI in patients with epilepsy. Neuroimage.

[B31] Lawson JA, Cook MJ, Vogrin S, Litewka L, Strong D, Bleasel AF, Bye AM (2002). Clinical, EEG, and quantitative MRI differences in pediatric frontal and temporal lobe epilepsy. Neurology.

[B32] Briellmann RS, Jackson GD, Kalnins R, Berkovic SF (1998). Hemicranial volume deficits in patients with temporal lobe epilepsy with and without hippocampal sclerosis. Epilepsia.

[B33] Bouilleret V, Dupont S, Spelle L, Baulac M, Samson Y, Semah F (2002). Insular cortex involvement in mesiotemporal lobe epilepsy: a positron emission tomography study. Ann Neurol.

[B34] Kim YK, Lee DS, Lee SK, Kim SK, Chung CK, Chang KH, Choi KY, Chung JK, Lee MC (2003). Differential features of metabolic abnormalities between medial and lateral temporal lobe epilepsy: quantitative analysis of (18)F-FDG PET using SPM. J Nucl Med.

[B35] Arnold S, Schlaug G, Niemann H, Ebner A, Luders H, Witte OW, Seitz RJ (1996). Topography of interictal glucose hypometabolism in unilateral mesiotemporal epilepsy. Neurology.

[B36] Engel J, Brown WJ, Kuhl DE, Phelps ME, Mazziotta JC, Crandall PH (1982). Pathological findings underlying focal temporal lobe hypometabolism in partial epilepsy. Ann Neurol.

[B37] Chassoux F, Semah F, Bouilleret V, Landre E, Devaux B, Turak B, Nataf F, Roux FX (2004). Metabolic changes and electro-clinical patterns in mesio-temporal lobe epilepsy: a correlative study. Brain.

[B38] Koutroumanidis M, Binnie CD, Elwes RD, Polkey CE, Seed P, Alarcon G, Cox T, Barrington S, Marsden P, Maisey MN, Panayiotopoulos CP (1998). Interictal regional slow activity in temporal lobe epilepsy correlates with lateral temporal hypometabolism as imaged with 18FDG PET: neurophysiological and metabolic implications. J Neurol Neurosurg Psychiatry.

[B39] Nelissen N, van Paesschen W, Baete K, Van Laere K, Palmini A, Van Billoen H, Dupont P (2006). Correlations of interictal FDG-PET metabolism and ictal SPECT perfusion changes in human temporal lobe epilepsy with hippocampal sclerosis. Neuroimage.

[B40] Foldvary N, Lee N, Hanson MW, Coleman RE, Hulette CM, Friedman AH, Bej MD, Radtke RA (1999). Correlation of hippocampal neuronal density and FDG-PET in mesial temporal lobe epilepsy. Epilepsia.

[B41] Matheja P, Kuwert T, Ludemann P, Weckesser M, Kellinghaus C, Schuierer G, Diehl B, Ringelstein EB, Schober O (2001). Temporal hypometabolism at the onset of cryptogenic temporal lobe epilepsy. Eur J Nucl Med.

[B42] Theodore WH, Kelley K, Toczek MT, Gaillard WD (2004). Epilepsy duration, febrile seizures, and cerebral glucose metabolism. Epilepsia.

[B43] Spanaki MV, Kopylev L, Liow K, DeCarli C, Fazilat S, Gaillard WD, Theodore WH (2000). Relationship of seizure frequency to hippocampus volume and metabolism in temporal lobe epilepsy. Epilepsia.

[B44] Dlugos DJ, Jaggi J, O'Connor WM, Ding XS, Reivich M, O'Connor MJ, Sperling MR (1999). Hippocampal cell density and subcortical metabolism in temporal lobe epilepsy. Epilepsia.

[B45] Theodore WH, Gaillard WD, De Carli C, Bhatia S, Hatta J (2001). Hippocampal volume and glucose metabolism in temporal lobe epileptic foci. Epilepsia.

[B46] Vinton AB, Carne R, Hicks RJ, Desmond PM, Kilpatrick C, Kaye AH, O'Brien TJ (2007). The extent of resection of FDG-PET hypometabolism relates to outcome of temporal lobectomy. Brain.

